# Overexpression of estrogen receptor β inhibits cellular functions of human hepatic stellate cells and promotes the anti-fibrosis effect of calycosin via inhibiting STAT3 phosphorylation

**DOI:** 10.1186/s40360-022-00617-y

**Published:** 2022-10-07

**Authors:** Yaxin Wang, Canyan Wu, Jiahui Zhou, Haiming Fang, Jiajia Wang

**Affiliations:** 1 Department of Pharmacology, School of Basic Medical Sciences of Anhui Medical University, NO.81 Meishan Road, Hefei, 230032 Anhui Province China; 2 Department of Gastroenterology, the Second Hospital of Anhui Medical University, NO.678 Furong Road, Hefei, 230601 Anhui Province China

**Keywords:** Estrogen receptor β, Calycosin, Liver fibrosis, Hepatic stellate cell, JAK/STAT

## Abstract

**Background:**

Estrogen receptor β (ERβ) is the major ER subtype in hepatic stellate cells (HSCs). Previously we reported phytoestrogen calycosin suppressed liver fibrosis progression and inhibited HSC-T6 cell functions, suggesting the effects may be related to ERβ. Here, we explore the effect of overexpressed ERβ on human HSCs and the role of ERβ in pharmacological action of calycosin.

**Methods:**

LX-2 cells were transfected with lentivirus to overexpress ERβ. In the presence or absence of overexpressed ERβ, the effects of ERβ and calycosin on proliferation, migration, activation, collagen production and degradation of TGF-β1-induced LX-2 cells and the role of ERβ in the inhibition effect of calycosin were investigated. LX-2 cells overexpressed with ERβ or treated with ER non-selective antagonist ICI182,780 were used to investigate the regulation of ERβ on JAK2/STAT3 signaling pathway. CCK-8 method was used to screen effective doses of calycosin and investigate cell proliferation. The cell migration was detected by transwell chamber assay. The expression of α-SMA was detected by immunofluorescence and western blot. The protein expressions of Col-I, MMP1, TIMP1, JAK2, p-JAK2, STAT3 and p-STAT3 were detected by western blot.

**Results:**

ERβ overexpressed lentivirus was successfully transfected into LX-2 cells with high efficiency. Overexpressed ERβ or calycosin alone inhibited the TGF-β1-induced LX-2 cell proliferation and migration, downregulated the protein expressions of α-SMA, Col-I, TIMP-1, p-STAT3 and upregulated MMP-1. Both overexpressed ERβ and calycosin had no significant effect on JAK2, p-JAK2 and STAT3 expressions. ERβ overexpression further enhanced the above effects of calycosin. However, after the cells were treated with ICI182,780, downregulation of STAT3 phosphorylation induced by calycosin was reversed.

**Conclusions:**

ERβ mediated the inhibition of major functions of LX-2 cell possibly by inhibiting the phosphorylation of STAT3, and was an important pathway through which calycosin exerted anti-liver fibrosis effect.

**Graphical Abstract:**

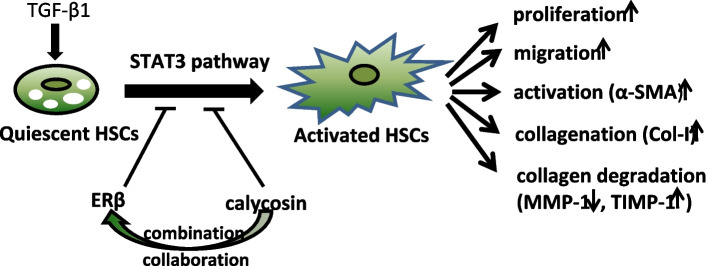

## Introduction

Liver fibrosis is a complex inflammatory and fibrogenic response generated as a result of chronic liver injury due to various factors, resulting in abnormal wound healing and excessive accumulation of extracellular matrix (ECM) [1]. Liver fibrosis is a serious public health problem with a high risk of progression to advanced liver cirrhosis and hepatocellular carcinoma (HCC) if the insult is not eliminated. Thankfully, if the underlying cause of liver injury is alleviated or cured, liver fibrosis may be reversible at early stages [2]. Epidemiological evidences suggest that the progression of hepatic fibrogenesis and carciogenesis is greater in men and postmenopausal women than in premenopausal women, and liver cirrhosis appears to progress more rapidly in males than in females. This sex-associated differences may be attributed at least in part to the lower production of estrogen and the reduced response to the action of estrogen (E2) [3]. E2, also known as 17beta-estradiol or estradiol, is the predominant biologically active form of circulating estrogen and passively diffuses across cell membranes to target tissues in plasma via sex hormone-binding globulin. E2 binds to the traditional E2 receptors (ERs), including estrogen receptor alpha (ERα), and estrogen receptor beta (ERβ), as well as the more recently identified G-protein-coupled ER1 (GPER1, also known as GPR30), and exerts both genomic and non-genomic actions [4]. Variant ERs are expressed to a greater extent in male patients with chronic liver disease than in females. Interestingly, we note that ERβ is highly expressed in hepatic stellate cells (HSCs), the key forming cells of hepatic fibrosis, while ERα is rather low or even not expressed [5]. The antifibrogenic effects of estrogen were mediated by ERβ but not ERα or GPER. The ERβ selective agonist hold the key to produce protective effects and fibrosuppressive effect of estrogens on liver fibrosis, while reducing undesired side effects [6]. Transgenic mouse model found that the liver was actually the most responsive organ to E2 signaling [7]. Animal experiments and clinical trials have provided consistent evidences for the protective effect of endogenous and exogenous estrogen on liver fibrosis [8]. However, long-term administration of exogenous estrogen is associated with a wide range of potential risks including malignancies, feminization, and thrombus formation due to its low selectivity to target organs [9, 10]. Therefore, it is crucial to discover and develop alternative estrogen substitutes that selectively regulate ERβ activity and may open up new avenues for the prevention and treatment of chronic liver disease.

Traditional Chinese medicine (TCM) has the encouraging potential to revert hepatic fibrosis. Radix Astragali (Huangqi), an edible TCM, has been commonly used as one of the primary tonic herbs for health care in China and East Asia for thousands of years. Radix Astragali contains multiple active components, including calycosin, saponins, polysaccharides, astragalosides, and other isoflavonoids. Calycosin is a phytoestrogen with similar structure to mammalian estrogens. Pharmacological studies and clinical practices have proven its effects on antioxidative free radicals, anti-hepatic damage, regulation of hepatocyte glucose uptake disorder, inhibition of hepatocellular carcinoma [11–14]. As phytoestrogens, calycosin inhibits the growth of various tumors through ERβ-mediated signaling pathway. Our previous studies revealed the anti-liver fibrosis effect of calycosin in CCl_4_-induced liver fibrosis in mice and its inhibition effect on the proliferation, migration and activation of HSC-T6 cells [15, 16]. JAK/STAT is a key signaling pathway in a variety of cellular activities, including proliferation, differentiation, migration, apoptosis and cell communication, as well as complex biological processes such as inflammation, immune response and cancer development [17]. The JAK family of kinases consists of JAK1, JAK2, JAK3 and TYK2. The STAT protein family consists of seven members encoded by different genes: STAT1, STAT2, STAT3, STAT4, STAT6, and the closely related STAT5A and STAT5B [18]. Activation of JAK2/STAT3 signaling pathway is considered as one of the most distinguished hallmarks of HSC activation and this pathway is also an ideal therapeutic strategy against chronic liver disease [19]. In our in vivo studies, we found that calycosin could increase ERβ protein expressions (but not ERα), JAK2 and STAT3 mRNA expressions, the ratio of p-JAK2/JAK2 and p-STAT3/STAT3, however, calycosin showed no significant effect on JAK2 and STAT3 protein expressions [15]. As is well known, the majority of breast cancers show overexpression of ERs, and hormone receptor status is the most significant predictive and prognostic biomarker of breast cancers. The development of drugs which target these hormone receptors has brought about significant improvement in survival for women with hormone receptor-positive breast cancers [20]. Delineating the absolute amounts and relative ratios of the different ERβ isoforms might have prognostic and therapeutic value, and contributes to better selection of optimal approaches for treatment of triple negative breast cancer [21]. However, the role of ERβ overexpression in liver fibrosis and in the anti-fibrosis effect of calycosin remains unclear. Thus, in this study, human hepatic stellate cell line (LX-2 cells) were transfected with lentivirus to overexpress ERβ. The effects of ERβ overexpression on activated LX-2 cells, the role of ERβ on the anti-fibrosis effect of calycosin and the regulation of JAK2/STAT3 signaling pathway were investigated.

## Materials and methods

### Cell culture and lentiviral transfection

LX-2 cells (DSMZ CVCL_5792) purchased from Shanghai Institute of Biochemistry and Cell Biology (Shanghai, China). The ERβ-overexpressing vector pHBLV-CMV-MCS-3FLAG-EF1- ZsGreen-T2A-PURO and the empty vector were purchased from HANBIO (Shanghai, China). LX-2 cells were cultured in DMEM (Hyclone, USA) with 10% fetal bovine serum (FBS, Ever Green, China) and were maintained in a humidified incubator at 37 °C with 5% CO_2_. Lentivirus transfection was operated according to the manufacturer’s instructions. For cell transfection, LX-2 cells were transferred into 6-well plates at the density of 2 × 10^5^ cells/well. After overnight culturing, LX-2 cells were transfected with ERβ overexpressing lentivirus for 48 h or 72 h. To determine the optimal multiplicity of infection (MOI), the lentivirus with MOI of 10, 20, 30 and 40 was respectively added to each well according to the instructions. The expression of green fluorescent protein in the transfected cells was observed by fluorescence microscope (Olympus, Japan) 24 h and 48 h after transfection. Stable transfected LX-2 cells were screened using fresh medium containing 2 μg/mL purinomycin for 24 h. Overexpression of ERβ was identified by determination of mRNA and protein expression.

### LX-2 cell proliferation assay

The effects of calycosin (Phytomarker Ltd., Tianjing, China) on cell proliferation were evaluated by the cell counting kit-8 (CCK-8) assay (Beyotime, Shanghai, China) according to the manufacturer’s instructions. Quiescent LX-2 cells were first treated with calycosin (25, 50, 100, 200, 300, 600 μM) to investigate the cytotoxicity and safe dose range of calycosin. Then, LX-2 cells were pretreated with safe concentrations of calycosin (25, 50, 100, 200 μM) or E2 (1μM, Shanghai yuanye biotechnology Co., Ltd., Shanghai, China) for 12 h and then stimulated with 10 ng/ml TGF-β1 (R&D Systems, Minneapolis, MN, USA) for 12 h.

LX-2 cells or transfected cells (MOI = 40) were seeded into 96-well plates at a density of 5 × 10^3^ cells/well at 24 h, 48 h and 72 h. TGF-β1 (10 ng/ml) was added into cells (transfected for 72 h) for another 12 h to show the effect of TGF-β1 on proliferation of ERβ overexpressing cells. After incubated with serum-free media for 24 h, LX-2 cells or transfected cells were cultured in DMEM containing calycosin dissolved in dimethyl sulfoxide (DMSO, Beyotime, Shanghai, China) or E2 for 12 h. TGF-β1 with final concentration of 10 ng/ml was then added for another 12 h. At the end of the treatment, the medium was replaced with 90 μl DMEM supplemented with 10% FBS, and 10 μl CCK-8 was added into each well, then cells were incubated for an additional 3 h. Absorbance was detected by a Multiskan GO (Thermo, USA) at 450 nm.

### Cell migration assay

According to our previous method [16], the migration ability of LX-2 and transfected cells was inspected by 24-well corning transwell chambers (8.0 μm pore size polycarbonate membrane, Corning Costar, USA). Cells were washed with PBS after digestion and resuspended in serum-free DMEM. Then 2 × 10^4^ cells were seeded into the upper chambers and 0.6 ml DMEM containing 10% FBS was added to the bottom chambers. After 12 h culturing, cells were treated with calycosin (200 μM) or E_2_ (1μM) for 24 h, and then 10 ng/ml TGF-β1 was added to upper chambers. After 12 h of incubation, all unmigrated cells were removed from the upper surface of the filters and the migrated cells were fixed and stained with crystal violet solution (Shanghai yuanye Bio-Technology, Shanghai, China). All the stained cells were counted in five different fields under a 400-fold microscope (Olympus, Japan).

### Immunofluorescence

According to our previous method [16], LX-2 cells (2.5 × 10^4^ cells/well) with or without ERβ overexpression were seeded onto the cover glass on a 24-well cell culture plate. After starving overnight in DMEM containing 2% FBS, except cells in control and vechile groups, all cells were stimulated by 10 ng/ml TGF-β1 for 12 h with or without pretreatment of calycosin (200 μM) or E_2_ (1μM) for 24 h. Then cells were washed with PBS three times and fixed in 4% paraformaldehyde (Beyotime, Shanghai, China) for 15 min at room temperature. After twice washes, cells were incubated for 10 min with PBS containing 0.2% Triton X-100 (Sangon Biotech, Shanghai, China). Cells were washed by PBS twice again and then blocked in PBS containing 5% BSA for 1 h at room temperature. Afterwards, the cells were incubated overnight at 4 °C with anti-α-SMA (1:300) primary antibody (Bioss, China) and then gently washed with PBS 3 times and further incubated with TRITC-conjugated secondary antibodies (1:50, ZSGB-BIO, China) for 1 h at room temperature in dark. Finally, cells were washed and then incubated with DAPI (1 μg/mL, Beyotime, Shanghai, China) for another 15 min at room temperature. The co-localization expression was visualized under confocal microscope (Nikon A1, Japan).

### Quantitative real-time PCR

Quantitative real-time PCR (qRT-PCR) was carried out according to our previous methods [15]. Total RNA extracted from cells were lysed by TRIzol reagent (Invitrogen, Carlsbad, CA) according to the manufacturer’s instructions. Then the synthesis of cDNA was performed using the primeScript™ RT reagent kit (Takara Biotechnology, Shiga, Japan) and quantitative PCR was performed on a 7500 Real Time PCR system (Applied Biosystems, Foster City, CA, USA) with SYBR® premix Ex Taq™ (Takara, Cambridge, MA, USA) according to the instructions. Targeted cDNAs were amplified for 95 °C, 30 s (1 cycle); 95 °C, 5 s; and 60 °C, 34 s (40 cycles). The primers used for PCR amplification were as follows: ERβ (forward 5′-GAATGGTCAAGTGTGGATCCAGGAG-3′; reverse 5′-CTCCATCCAGCAGTT-TCCAAGAGG-3′) and β-actin (forward 5′-TCCTCCTGAGCGCAAGTACTCT-3′; reverse 5′-GCTCAGTAACAGTCCGCCTAGAA-3′). The relative level of ERβ mRNA was quantified by the comparative threshold cycle (^ΔΔ^Ct) method.

### Western blotting analysis

Quiescent LX-2 cells were pretreated with calycosin (200 μM) or E_2_ (1μM) for 24 h and then TGF-β1 (10 ng/ml) was added for 12 h. LX-2 cells were washed twice with cold PBS and lysed in RIPA (Beyotime, Shanghai, China) containing 1% PMSF (Beyotime, Shanghai, China) and 1% phosphatase inhibitor (New Cell& Molecular Biotech, Suzhou, China) for 30 min on ice. Cells were scraped from the plate and centrifuged at 12000 r/min for 30 min at 4 °C. The protein concentrations were measured by BCA assay kit (Beyotime, China) according to the manufacturer’s protocol. Then samples were electrophoresed by 10% SDS-PAGE gels and subsequently transferred to 0.45 μm PVDF membranes (Millipore, USA) at 200 mA for 1 h. The membranes were blocked with 5% nonfat dried milk (Beyotime, Shanghai, China) in Tris-buffered solution (TBST, pH 7.6, 0.05% Tween 20) at room temperature for 1 h. Then the membranes were washed with TBST 3 times for 10 min each. According to the position indicated by marker, the membranes with target proteins were cut off, and thus the images with adequate length were not obtained. The membranes were incubated separately at 4 °C overnight in the following diluted primary antibodies: mouse anti-ERβ antibody (1:1000, Abcam, UK), rabbit anti-α-SMA antibody (1:1000, Bioss, China), rabbit anti-Col I antibody (1:1000, Bioss, China), rabbit anti-MMP1 (1:1000, Bioss, China), rabbit anti-TIMP1 (1:1000, Bioss, China), mouse anti-JAK2 antibody (1:1000, Bioss, China), rabbit anti-p-JAK2 antibody (1:1000, Affinity, America), rabbit anti-STAT3 antibody (1:1000, Bioss, China), rabbit anti-p-STAT3 antibody (1:1000, Bioss, China), rabbit anti-GAPDH antibody (1:2000, Bioss, China), mouse anti-β-actin antibody (1:1000, ZSGB-BIO, China). After 3 washes, membranes were incubated in diluted secondary antibodies (anti-mouse IgG-HRP 1:10000, anti-rabbit IgG-HRP 1:10000, ZSGB-BIO, China) at room temperature for 1 h. The proteins on the PVDF membranes were detected with electrochemiluminescence (ECL) western blot detection reagents (Bridgen, China). Image J was used to analyze the grayscale of stripes.

### Statistical analysis

Data were represented as mean ± SD for at least three independent experiments. The data were analyzed by Student t test for paired comparisons using SPSS 23.0 and Graphpad prism 7.0 software. *P* < 0.05 was considered statistically significant. The significance between groups was marked on the graphs.

## Results

### Transfection efficiency of ERβ overexpression in LX-2 cells

**Fig. 1:**
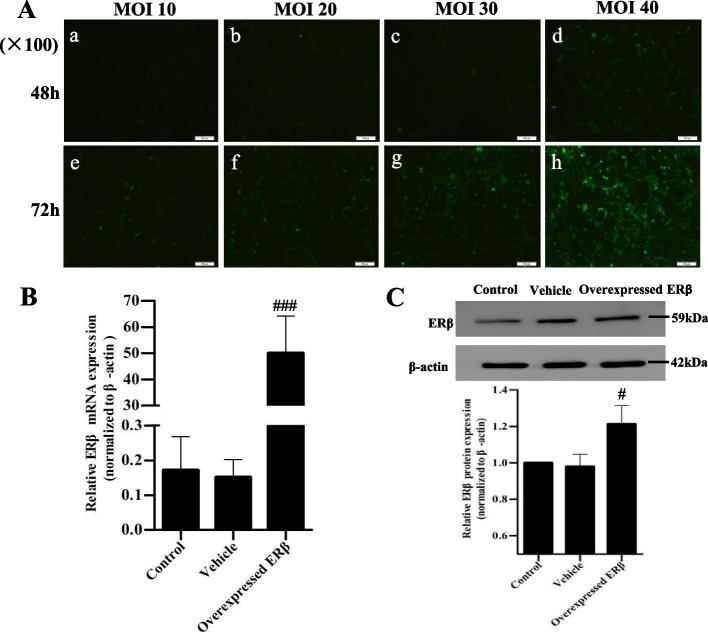
Efficiency of lentivirus infection and overexpression of ERβ in LX-2 cells. **A** Lentivirus transfection efficiency of LX-2 cells observed by fluorescence microscope (× 100). To determine the optimal transfection conditions, the ERβ-overexpressed lentivirus with multiplicity of infection (MOI) of 10, 20, 30 and 40 was transfected to LX-2 cells for 48 h (**a-d**) and 72 h (**e-f**), respectively. The positive transfected cells were shown in green fluorescence. Stable transfected cells were screened using 2 μg/mL purinomycin for 24 h. **B** Relative mRNA expression of ERβ detected by quantitative real-time PCR. Intensity of ERβ was standardized to that of β-actin. Data were shown as mean ± SD of 3 independent experiments. **C** The overexpression of ERβ protein in LX-2 cells was measured by western blot. The β-actin was used for normalization. The experiment was performed in triplicate and repeated three times. ^#^ *p* < 0.05, ^###^*p* < 0.001 vs vehicle group

To detect the transfection efficiency of transfected cells, cell morphology and number were observed by fluorescence microscope, and the mRNA and protein expressions of ERβ were detected by qRT-PCR and western blotting. The positive transfected cells showed green fluorescence (Fig. 1A). Under the optimal experimental conditions of MOI 40 and infection time 72 h, the positive transfected cells accounted for more than 80% of the total cells. ERβ mRNA and protein levels were significantly increased in the transfected cells (Fig. 1B, C), indicating LX-2 cells successfully overexpressed ERβ.

### ERβ overexpression enhanced the inhibitory effect of calycosin on TGF-β1 induced LX-2 cells proliferation

**Fig. 2:**
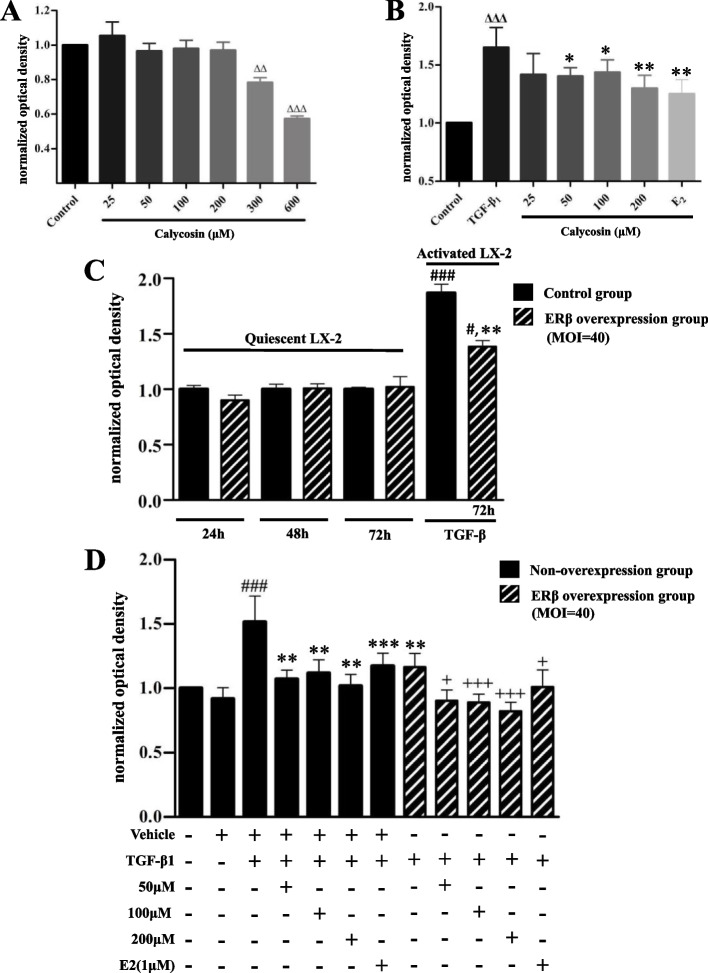
The effect of ERβ overexpression and calycosin on TGF-β1-induced LX-2 cells proliferation. Cell counting kit-8 (CCK-8) assay was used to select the safe concentration range of calycosin on normal LX-2 cells **(A)** and the effective concentration range of calycosin on TGF-β1-induced LX-2 cells **(B)**. **C** LX-2 cells and transfected cells (MOI = 40) were cultured for 24 h, 48 h and 72 h to show the effect of ERβ overexpression alone on cell proliferation. LX-2 cells transfected for 72 h were treated with TGF-β1 (10 ng/ml) for another 12 h to investigate the effect of TGF-β1 on proliferation of ERβ overexpressing cells. **D** The effect of ERβ, calycosin (50, 100, 200 μM) and E2 (1μM) on proliferation of TGF-β1-induced LX-2 cells were determined in the presence or absence of ERβ overexpression. In all the TGF-β1 induced cell groups, cells were pretreated with calycosin or E2 for 24 h and then stimulated with 10 ng/ml TGF-β1 for 12 h. Data were normalized in relation to the mean value of the control group and shown as mean ± SD of three independent experiments. ^ΔΔ^*p* < 0.01, ^ΔΔΔ^*p* < 0.001 vs control group; ^#^*p* < 0.05, ^###^*p* < 0.001 vs corresponding overexpression or vehicle group; **p < 0.01, ***p < 0.001 vs TGF-β1 group; ^**+**^*p* < 0.05, ^**+++**^*p* < 0.001 vs the corresponding treatment groups without ERβ overexpression

The safe concentration selection and cytotoxicity of calycosin were assessed on normal LX-2 cells. Compared with the control group, the calycosin with concentration in the range of 25–200 μM had no significant effect on cell proliferation. However, when the concentration is higher than 300 μM, cells proliferation were significantly inhibited (*p* < 0.05, Fig. 2A). The result suggested that calycosin was safe to LX-2 cells in the range of 25-200 μM, and produced cytotoxicity in high concentration. As shown in Fig. 2B, TGF-β1 treatment stimulated quiescent LX-2 cells and significantly promoted proliferation compared with the control group (*p* < 0.001). When TGF-β1-induced LX-2 cells were pretreated with different dose of calycosin (50-200 μM), the cell proliferation was significantly inhibited (*p* < 0.05). Calycosin had no significant effect on quiescent LX-2 cells, but inhibited the proliferation of TGF-β1-induced cells, suggesting that calycosin had therapeutic effects on activated LX-2 cells.

In Fig. 2C, ERβ overexpression alone showed no affect in the proliferation of resting cells, but it significantly inhibited the proliferation of cells activated by TGF-β1. Compared with corresponding overexpressed group, TGF-β1 promoted the proliferation of ERβ overexpressing cells which were transfected for 72 h.

Furthermore, we investigated the role of ERβ in TGF-β1-induced LX-2 cells and the effect of calycosin on the proliferation of ERβ overexpressed LX-2 cells by CCK-8 assay. As shown in Fig. 2D, ERβ overexpression alone inhibited TGF-β1-induced LX-2 cell proliferation. LX-2 cells induced by TGF-β1 were treated with different doses of calycosin (50-200 μM), and calycosin exhibited significantly inhibitory effect on cell proliferation. In ERβ overexpression groups, the same concentration (50-200 μM) of calycosin showed stronger inhibitory effect on cell proliferation than that in TGF-β1-induced LX-2 cells without ERβ overexpression, indicating that ERβ overexpression enhanced the inhibition effects of calycosin. ERβ might play a negative role in regulating the proliferation of LX-2 cells.

### ERβ overexpression enhanced the inhibitory effect of calycosin on the migration of TGF-β1-induced LX-2 cells

**Fig. 3:**
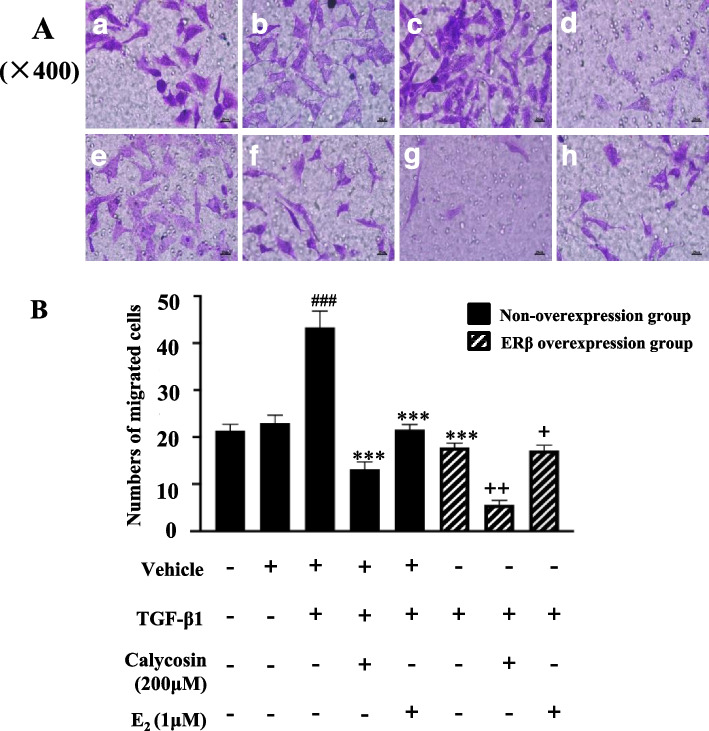
Inhibitory effect of ERβ overexpression and calycosin on the migration of TGF-β1-induced LX-2 cells. **A** Representative cell fields for each group (× 400). Cell transwell migration assay was applied to cells in control group (**a**), vehicle group (**b**), TGF-β1 model group (**c**), TGF-β1 + calycosin group (**d**), TGF-β1 + E_2_ group (**e**), TGF-β1 + ERβ overexpression group (**f**), TGF-β1 + ERβ overexpression+ calycosin group (**g**), and TGF-β1 + ERβ overexpression+E_2_ group (**h**). Cells were treated with calycosin (200 μM) or E_2_ (1μM) for 24 h, and then 10 ng/ml TGF-β1 was added to upper chambers. After 12 h of incubation, all unmigrated cells were removed from the upper surface of the filters and the migrated cells were fixed and stained with crystal violet solution . **B** The average number of invasive cells per field in each group. Numerical representation of the data was obtained by counting the average number of stained cells from five random fields of each group using a 400-fold microscope. Data are shown as mean ± SD of three independent experiments. ^###^*p* < 0.001 vs vehicle group; ***p < 0.001 vs TGF-β1 group; ^+^*p* < 0.05, ^++^*p* < 0.01 vs the corresponding treatment groups without ERβ overexpression

Transwell migration assay showed that TGF-β1 stimulation significantly increased LX-2 cells migration compared to the control group (*p* < 0.001). Compared with the model group, treatment of calycosin and ERβ overexpression both significantly reduced the migration of LX-2 cells induced by TGF-β1 (*p* < 0.001). In the ERβ overexpression group, the migration number of LX-2 cells treated with calycosin or E2 was significantly lower than that of the cells without ERβ overexpression (*p* < 0.05), indicating ERβ overexpression significantly enhanced the inhibitory effect of calycosin on LX-2 cell migration (Fig. 3).

### Effects of ERβ overexpression and calycosin on α-SMA, col-I, MMP-1 and TIMP-1 expressions in LX-2 cells

**Fig. 4:**
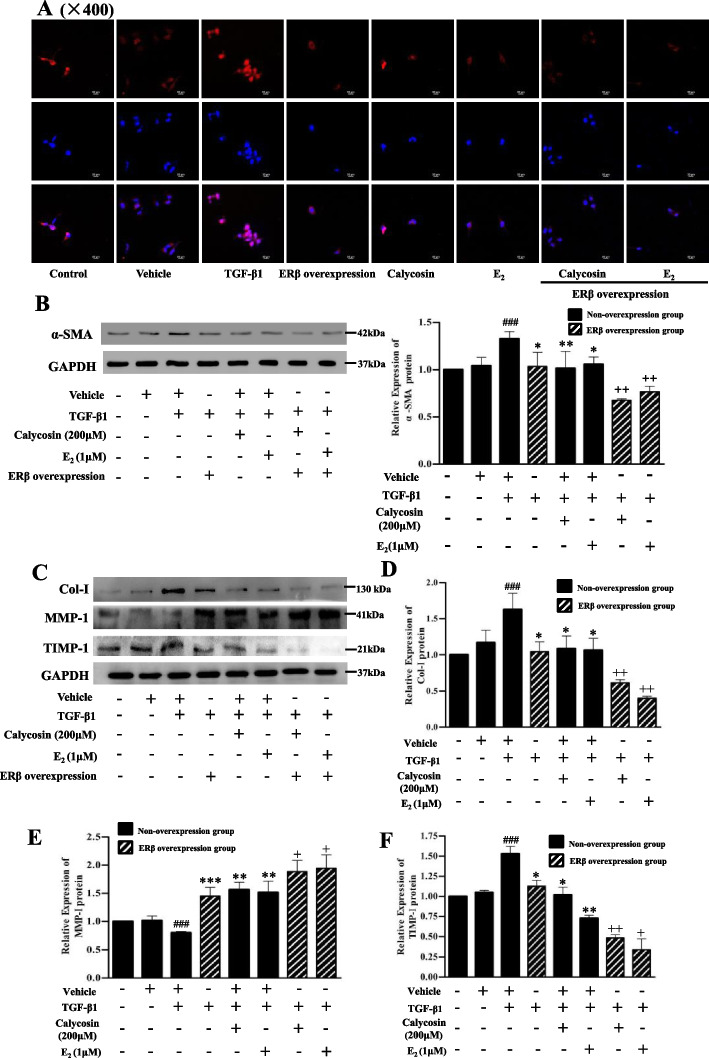
The effects of ERβ overexpression and calycosin on α-SMA, Col-I, MMP-1 and TIMP-1 expressions in TGF-β1-induced LX-2 cells. **A** Immunofluorescence images of α-SMA protein (red) in LX-2 cells with original magnification, × 400. Cell nuclei (blue) were stained with DAPI. Except cells in control and vechile groups, cells with/without ERβ overexpression were stimulated by 10 ng/ml TGF-β1 for 12 h with or without pretreatment of calycosin (200 μM) or E2 (1μM) for 24 h. (**B-F**) Representative blots and relative quantification analysis of α-SMA (**B**), Col-I (**D**), MMP-1 (**E**) and TIMP-1 (**F**) protein expressions detected by western blot. GAPDH was used as internal control. Data are shown as mean ± SD of three independent experiments. ^###^*p* < 0.001 vs vehicle group; *p < 0.05, **p < 0.01, ***p < 0.001 vs TGF-β1 group; ^+^*p* < 0.05, ^++^*p* < 0.01 vs the corresponding treatment groups without ERβ overexpression

The expression of α-SMA in LX-2 cells was detected by immunofluorescence and western blotting respectively (Fig. 4A, B). The nucleus of LX-2 cells showed blue fluorescence and α-SMA protein showed red fluorescence. The red fluorescence in ERβ overexpressed cells were significantly decreased after the cells were treated with calycosin or E2 respectively. The overexpression of ERβ alone could also decreased the red fluorescence in LX-2 cells even without calycosin treatment (Fig. 4A). The protein expressions of collagen-I (Col-I), matrix metalloproteinase-1 (MMP-1) and tissue inhibitor of matrix metalloproteinase-1 (TIMP-1) were determined by western blotting. Compared with the vehicle group, TGF-β1 significantly up-regulated the protein expressions of α-SMA, Col-I and TIMP-1 and down-regulated MMP-1 in LX-2 cells (*p* < 0.001). Compared to the TGF-β1 group, ERβ overexpression group as well as the calycosin/E2 treatment groups with/without ERβ overexpression showed significant down-regulation of α-SMA, Col-I, TIMP-1 protein expressions and up-regulation of MMP-1 protein expression (Fig. 4B,C,D,E,F). Compared to the corresponding calycosin/E2 treatment groups without ERβ overexpression, α-SMA, Col-I, TIMP-1 protein expressions in ERβ-overexpressed calycosin/E2 treatment group were further down-regulated (*p* < 0.01, Fig. 4B,C,D,F), while MMP-1 protein expressions were further up-regulated (*p* < 0.05, Fig. 4E). The results indicated that ERβ overexpression and calycosin alone could markedly regulate the cell activation, collagen formation and degradation in LX-2 cells, and ERβ overexpression enhanced all the above effects of calycosin.

### Effects of ERβ overexpression and calycosin on the JAK-STAT signaling pathway in LX-2 cells

**Fig. 5:**
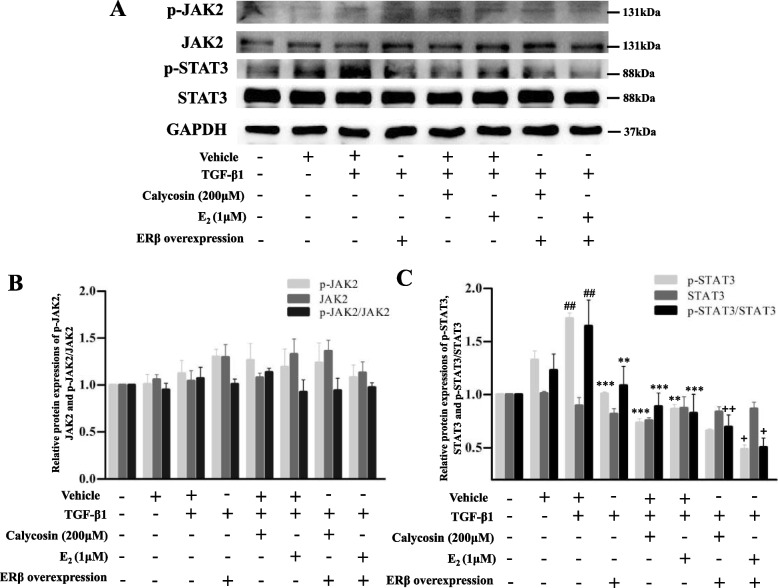
The effects of ERβ overexpression and calycosin on JAK2/STAT3 pathway in TGF-β1-induced LX-2 cells. **A** Representative blots of p-JAK2, JAK2, p-STAT3, STAT3 proteins detected by western blot. **B, C** Relative quantification analysis of p-JAK2, JAK2, p-STAT3, STAT3 protein expressions and the ratios of p-JAK2/ JAK2 and p-STAT3/STAT3. GAPDH was used as an internal control. Data are shown as mean ± SD of three independent experiments. ^##^*p* < 0.05 vs vehicle group; **p < 0.01, ***p < 0.001 vs TGF-β1 group; ^+^*p* < 0.05, ^++^*p* < 0.01 vs the corresponding treatment groups without ERβ overexpression

**Fig. 6:**
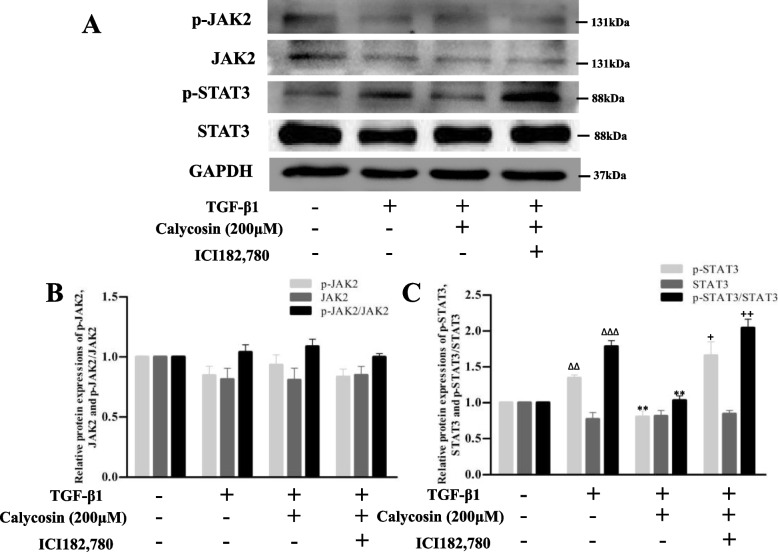
Effect of estrogen receptor antagonist ICI182,780 on JAK2/STAT3 pathway inhibited by calycosin in TGF-β1-induced LX-2 cells. **A** Representative blots of p-JAK2, JAK2, p-STAT3, STAT3 proteins detected by western blot. **B, C** Relative quantification analysis of p-JAK2, JAK2, p-STAT3, STAT3 protein expressions and the ratios of p-JAK2/ JAK2 and p-STAT3/STAT3. GAPDH was used as an internal control. Data are shown as mean ± SD of three independent experiments. ^ΔΔ^*p* < 0.01, ^ΔΔΔ^*p* < 0.001 vs control group; **p < 0.01 vs TGF-β1 group; ^+^*p* < 0.05, ^++^*p* < 0.01 vs the corresponding treatment groups without ERβ overexpression

As shown in Fig. 5, TGF-β1 up-regulated the protein expression of p-STAT3 compared with the vehicle group (*p* < 0.05). Compared with TGF-β1 model group, p-STAT3, the ratio of p-STAT3/STAT3 were significantly down-regulated in the ERβ overexpression group and calycosin/E2 treatment groups, while there were no significant changes in p-JAK2, JAK2, or STAT3 protein levels. We noted that the overexpression of ERβ alone could down-regulate STAT3 phosphorylation level (*p* < 0.001), but had no affect on JAK2. When calycosin/E2 were treated to ERβ overexpressed LX-2 cells, the expressions of p-JAK2, JAK2, STAT3 proteins still showed no significant changes, however, the ratios of p-STAT3/STAT3 were further decreased than that of corresponding ERβ non-overexpression groups. Furthermore, ER non-selective antagonist ICI182,780 was used to further explore. As shown in Fig. 6, compared with the calycosin group, p-JAK2, JAK2 and STAT3 protein expressions in ICI182,780 treated cells still had no significant changes, but p-STAT3 protein expression was significantly increased (*p* < 0.05), indicating ICI182,780 reversed the inhibitory effect of calycosin on STAT3 phosphorylation. In the study, the treatment of ERβ overexpression, calycosin and ER antagonist in LX-2 cells only showed significant effect on STAT3 phosphorylation, suggesting that ERβ overexpression may exert its effect and enhance inhibitory effect of calycosin by inhibiting STAT3 phosphorylation.

## Discussion

With the ever-evolving understanding of the cellular and molecular mechanisms of liver fibrosis, the therapeutics focus on either controlling the initial trigger of fibrogenesis, interrupting receptor ligand interactions and other intracellular communications, inhibiting fibrogenesis, or even reverse liver fibrosis [2]. Emerging evidences have shown that the activation and proliferation of HSCs is regarded as the pivotal link to liver fibrosis. The quiescent HSCs are activated and then transformed into myofifibroblasts when liver injury occurs. Abnormal wound healing responses lead to excessive accumulation of ECM when chronic liver injury persists [22]. The quality, quantity, composition and arrangement of liver ECM components changed significantly, mainly due to the increase of collagen fibers, of which Col-I was the dominant collagen fiber. MMPs and its inhibitors TIMPs are key enzymes in the dynamic process of controlling the balance between deposition and degradation of ECM in liver fibrosis.

In the present study, the overexpressed ERβ lentivirus was successfully transfected into LX-2 cells and activated human HSCs model has been successfully established by TGF-β1 stimulation. TGF-β stimulation markedly promoted the proliferation, migration, and activation of LX-2 cells, up-regulated the expressions of α-SMA, Col-I, TIMP-1 and down-regulated MMP-1 expression in LX-2 cells. Furthermore, TGF-β1 also promoted the proliferation of ERβ overexpressing cells, however, this effect was attenuated compared with that on non-overexpressed cells. ERβ overexpression and calycosin negatively regulated the above effects on TGF-β1-induced LX-2 cells, respectively. ERβ overexpression combined with calycosin treatment played a synergistic role on the above effects. Combined with our previous in vitro and in vivo results [15, 16], we further confirmed that calycosin inhibits HSCs functions through ERβ.

Interestingly, the migration inhibition of calycosin on LX-2 cells seems to be more pronounced, ERβ overexpression also enhanced this effect. However, mitomycin C was not pretreated to cells to exclude cell proliferation interference in migration assay, so our results cannot exclude the interference of cell proliferation on migration. In fact, we’ve found that both ERβ overexpression and calycosin inhibited TGF-β1-induced LX-2 cells proliferation (Fig. 2D). It is therefore possible that the inhibition of migration is partly due to the inhibition of proliferation.

Our results supported that calycosin exerts pharmacological effects by activating ERβ. Perhaps just as receptor agonists may cause downregulation of the corresponding receptor, calycosin was also found to down-regulate ERβ expression in our previous in vitro experiment [16]. This attracted our attention and further studies need to be conducted to determine whether the down-regulated receptors will affect the efficacy of the drug.

JAK has the ability to activate STATs. STAT3 is a valued member of the JAK/STAT signaling pathway and is closely related to the occurrence and development of liver fifibrosis caused by various factors [23]. To further confirm the involvement of JAK2/STAT3 in the possible molecular mechanisms, effects of ERβ overexpression and calycosin on JAK2/STAT3 signaling pathway were detected by western blotting. As a result, phosphorylation of STAT3 was inhibited by ERβ overexpression and/or calycosin in LX-2 cells. ERβ overexpression promoted the inhibitory effect of calycosin on STAT3 phosphorylation, while deficiency of ERβ activation (antagonized by ICI182,780) reversed this inhibition effect. Therefore, we assumed that ERβ overexpression inhibited the activation and proliferation of human HSCs and promoted the anti-fibrosis effect of calycosin by inhibiting STAT3 phosphorylation.

The activity of calycosin is conducted through its interaction with ERs, which has been documented previously [24, 25]. It has been shown in various organs that inhibition of STAT3 reduces TGF-β1-induced fibroblast activation and hence relieves tissue fibrosis [26–28]. The contribution of our study to the understanding of the antifibrotic effect of calycosin as a candidate drug on HSC-mediated liver fibrosis is incremental. However, in contrast to ERβ -mediated action, ERα was found to enhance the phospholyation and acetylation of STAT3 in breast cancer cells [29]. This is consistent with the claim that ERα and ERβ generally play opposite roles in many tissues. On the other hand, we also found that ERβ overexpression and calycosin had no significant affect on JAK2 in LX-2 cells. We speculated that the synergistic effect of ERβ overexpression and calycosin on STAT3 activity may be independent of JAK2 in LX-2 cells. Moreover, there may be endogenous ligands or regulatory factors that enable ERβ to cause changes in cellular function when ERβ is overexpressed alone.

However, liver fibrosis is a complex pathologic process including several pathways. For instance, as is well known, TGF-β1 contributes to the evolution of fibrosis through activating Smads proteins. Subsequently, Smads alters the transcription of multiple genes causing fibrosis. We assumed that the ERβ overexpression could affect the expressions of multiple downstream genes related to liver fibrosis by activating STAT3. The specific mechanism remains to be further studied to clarify the synergistic mechanism of ERβ and calycosin against liver fibrosis.

## Conclusion

In summary, ERβ mediated the inhibition of major cellular functions of LX-2 cell possibly by inhibiting the phosphorylation of STAT3, and was an important pathway through which calycosin exert anti-liver fibrosis. Our research provided important insights into ERβ as a potential therapeutic target for liver fibrosis.

## Data Availability

The data supporting the conclusions of this article are included within the article. Additional data will be available upon request from corresponding author.

## Abbreviations

E2: Estrogen
ERβ: Estrogen receptor β
ECM: Extracellular matrix
α-SMA: Alpha smooth muscle actin
HCC: Hepatocellular carcinom
HSC: Hepatic stellate cell
Col-I: Collagen type-I
MMP: Matrix metalloproteinase
TIMP: Tissue inhibitors of metalloproteinases
JAK: Janus kinase
STAT: Signal transducer and activator of transcription
TGF-β1: Transforming growth factor β1
